# Low expression of organic anion-transporting polypeptide 1B3 predicts a poor prognosis in hepatocellular carcinoma

**DOI:** 10.1186/s12957-020-01891-y

**Published:** 2020-06-13

**Authors:** Shihan Chen, Kun Li, Jiayun Jiang, Xiaofei Wang, Yuelong Chai, Chang Zhang, Qingsong Deng, Ling Shuai, Kai Feng, Kuansheng Ma, Leida Zhang

**Affiliations:** 1 Department of Hepatobiliary Surgery, the First Affiliated Hospital of Army Medical University (Third Military Medical University), Chongqing, 400038 China; 2 Department of Hepatobiliary Surgery, the Second Affiliated Hospital of Chongqing Medical University, No. 74 Linjiang Road, Yuzhong District, Chongqing, 400010 China

**Keywords:** OATP1B3, Hepatocellular carcinoma, Prognosis marker, Survival

## Abstract

**Objective:**

To detect the expression level of organic anion-transporting polypeptide 1B3 (OATP1B3) in hepatocellular carcinoma (HCC) and to determine the relationship between OATP1B3 expression, clinicopathological features, and prognosis.

**Methods:**

Immunohistochemical (IHC) staining was performed to detect the expression of OATP1B3 in 131 HCC specimens and in 89 adjacent nontumorous tissues. Moreover, the expression levels of OATP1B3 in 30 pairs of tumor and matched adjacent nontumorous tissues were detected by quantitative real-time polymerase chain reaction, and 34 pairs of tumor and matched adjacent nontumorous tissues were detected by Western blotting. The χ^2^ test was applied to analyze the correlation between OATP1B3 expression and the clinical parameters of HCC patients. The prognostic value of OATP1B3 in HCC patients was estimated by Kaplan-Meier survival analysis and the Cox stepwise proportional hazards model.

**Results:**

Compared with that in adjacent nontumorous tissues (25.8%, 23/89), OATP1B3 expression was significantly downregulated in tumor tissues (59.5%, 78/131) (*P* < 0.0001). Moreover, OATP1B3 expression was markedly correlated with tumor size, recurrence, tumor differentiation, and tumor node metastasis (TNM) stage (*P* < 0.05 for each). However, age, sex, tumor capsule status, HBsAg, cirrhosis, tumor number, vascular invasion, and serum alpha fetoprotein were not associated with OATP1B3 expression. The overall survival (OS) and disease-free survival (DFS) of HCC patients who had high expression of OATP1B3 were significantly longer than those of patients with low expression (33.0% vs 12.9%, *P* = 0.001; 18.8% vs 5.3%, *P* < 0.0001). Cox multivariate analysis showed that OATP1B3, invasion, and TNM stage (*P* < 0.05 for each) were independent prognostic factors of OS in HCC patients and that OATP1B3 and TNM stage (both *P* < 0.05) were independent prognostic factors of DFS in HCC patients.

**Conclusions:**

The expression of OATP1B3 in HCC patients was significantly lower than that in adjacent nontumorous tissues. OATP1B3 expression may be a potential prognostic marker in HCC patients.

## Background

Liver cancer is one of the most common alimentary system malignancies and has high morbidity and mortality rates. According to the latest statistics, there are approximately 841,000 new cases of liver cancer and 782,000 related deaths worldwide every year [1, 2]. Hepatocellular carcinoma (HCC) is the most prominent subtype of primary liver cancer and accounts for 75–85% of all liver cancer cases [3]. HCC is often diagnosed at an advanced stage, as early symptoms are inconspicuous. Thus, it seriously endangers human health and life. Although some progress has been made in understanding the epidemiology, pathogenesis, diagnosis, and treatment of HCC in recent years [4], its high recurrence and metastasis rates still seriously affect patient survival. In clinical practice, the prognosis of HCC patients estimated by the Barcelona Clinic Liver Cancer (BCLC) and Tumor Node Metastasis (TNM) staging classification systems is often inaccurate [5]. This may arise because the tumor malignant phenotype is not included in these classification systems. Early effective intervention for patients with poor prognosis will be helpful in prolonging patient survival. Therefore, there is an urgent need to identify potential risk factors to evaluate the prognosis of patients with HCC.

Organic anion transporter polypeptide 1B3 (OATP1B3), also known as LST-2, OATP8, or SLC21A8, is a member of the solute-transporter superfamily [6, 7]. The coding gene is solute-carrier organic anion transporter 1B3 (SLCO1B3), located on human chromosome 12p12 [8]. The OATP1B3 protein contains 12 transmembrane domains with six extracellular loops and five intracellular loops. Both the carboxyl terminus and amino terminus of the OATP1B3 polypeptide chain are localized at the cytoplasmic side of cells [9]. OATP1B3 is predominantly expressed at the basolateral membrane of hepatocytes [6, 8]. Its main function is to transport a variety of endogenous and xenobiotic compounds (e.g., prostaglandins, steroid hormone conjugates, bilirubin, bile acids, thyroid hormones, methotrexate, paclitaxel) into hepatocytes for metabolism [8, 10]. Gadolinium ethoxybenzyl diethylenetriamine pentaacetic acid (Gd-EOB-DTPA)-enhanced magnetic resonance imaging (MRI) has been widely used to evaluate hepatocellular-specific characteristics in tumors in China since 2010. OATP1B3 is a key transporter for Gd-EOB-DTPA uptake [11] and is significantly associated with the enhancement ratios of HCCs on Gd-EOB-DTPA-enhanced MRI in the hepatobiliary phase [12, 13]. In recent years, a growing number of analysts have identified the role of OATP1B3 in tumors [14–21]. However, its expression and role in different tumor cells and tissues have been found to be different. Several previous studies revealed that the expression of OATP1B3 in colon, breast, pancreatic, and prostate cancer tissues is notably greater than that in adjacent nontumorous tissues [15–17, 22]. Lockhart et al. [15] reported that immunoreactivity of OATP1B3 was detectable in 56% of colorectal tumor samples and was correlated with improved 5-year survival in patients with poorly differentiated tumors. In contrast, Lee et al. [14] found that OATP1B3 has an important prosurvival/antiapoptotic effect in colon cancer cells. OATP1B3 significantly decreased the levels of p53 and substantially inactivated well-known p53 downstream target genes (NOXA, PUMA, and P21WAF1) in chemotherapy-treated cancer cells. In breast cancer, OATP1B3 expression was correlated with various pathological parameters and improved prognosis both in postmenopausal patients and estrogen receptor-positive patients [16]. In addition, Hamada et al. [17] showed that OATP1B3 can mediate the uptake of testosterone to support prostate cancer cell growth. Compared with patients carrying TT/AA and TG/GA haplotypes, the median survival time and survival rate of patients with the OATP1B3 334GG/699AA haplotype were significantly increased. In HCC, OATP1B3 expression was downregulated [9, 23, 24] and was significantly associated with multistep hepatocarcinogenesis [18]. These results indicate that OATP1B3 may play an important role in tumor biological function and has a substantial effect on patient survival outcome. To date, the impact of OATP1B3 expression on the prognosis of HCC patients has rarely been addressed. Therefore, the aims of this investigation were to explore the association between the expression of OATP1B3 and clinicopathological features in HCC patients and to assess the prognostic value of OATP1B3.

## Methods

### Tissue samples

Tumor tissue specimens were collected from 131 HCC patients who underwent hepatectomy from January 2012 to December 2012 in the First Affiliated Hospital of Army Medical University. In addition, 89 adjacent nontumorous tissue samples (less than 3 cm from the edge of the tumor) were also obtained. All patients were diagnosed with HCC by pathology. The patients had not received chemotherapy, radiotherapy, or other adjuvant treatments before surgery, and clinical data were complete for each patient. The grade of pathological differentiation was judged by the World Health Organization criteria. The clinical stage was classified according to the 2017 classification standard of the tumor node metastasis (TNM) system in the latest guidelines of the American Joint Committee on Cancer. The patients’ clinicopathological features are summarized in Table 1. The patients were followed up for 6 years. Thirty-four fresh HCC samples and adjacent nontumorous tissue samples were resected and placed in a freezing tube. These tubes were quickly frozen in liquid nitrogen and then transferred to a freezer at − 80 °C. This study was approved by the ethics committee of our hospital. Overall survival (OS) was defined as the time between the date of resection and date of death from any cause or the date of last follow-up. Disease-free survival (DFS) was defined as the time between the date of surgery and the date of the diagnosis of relapse.

**Table 1:** Association between OATP1B3 expression and clinicopathological features of HCC patients

Characteristics		***n***	OATP1B3 Low	OATP1B3 ***High***	***χ***^***2***^	***P***
HCC		131	78	53	24.236	< 0.0001
Nontumor liver		89	23	66		
Age (year)	≥ 50	56	33	23	0.015	0.902
	< 50	75	45	30		
Sex	Male	104	61	43	0.165	0.684
	Female	27	17	10		
Tumor size (cm)	≥ 5	88	59	29	6.266	0.012
	< 5	43	19	24		
TNM stage	I, II	50	19	31	15.578	< 0.0001
	III, IV	81	59	22		
Tumor differentiation	Well	9	0	9	17.716	< 0.0001
	Moderate	101	61	40		
	Poor	21	17	4		
Serum AFP	≥ 20 ng/mL	89	55	34	0.586	0.444
	< 20 ng/mL	42	23	19		
Recurrence	Yes	81	56	25	8.109	0.004
	No	50	22	28		
HBsAg	Positive	106	61	45	0.918	0.338
	Negative	25	17	8		
Tumor number	Single	87	47	40	3.275	0.07
	Multiple	44	31	13		
Liver cirrhosis	Yes	91	53	38	0.209	0.647
	No	40	25	15		
Invasion	Yes	58	36	22	0.276	0.599
	No	73	42	31		
Tumor capsule	Yes	45	27	18	0.006	0.938
	No	86	51	35		

*AFP* alpha fetoprotein, *OATP1B3* organic anion transporting polypeptide 1B3, *TNM stage* tumor node metastasis stage, *HCC* hepatocellular carcinoma

### Immunohistochemistry

Immunohistochemical (IHC) staining was performed using the streptavidin-peroxidase method. Tissue specimens were fixed with 10% formaldehyde and embedded in paraffin, and 4-μm thick sections were prepared. The slides were deparaffinized in xylene, hydrated in gradient ethanol, washed with phosphate-buffered saline (PBS), boiled in citrate solution (pH 6.0) for 10 min to retrieve the antigens in a microwave oven, and then cooled to room temperature. The slides were incubated with 3% hydrogen peroxide for 15 min to block endogenous peroxidase activity, followed by incubation with 10% goat serum (SP-9001, Zhongshan Biotechnology, China) for 10 min to reduce nonspecific reactions and incubation with a rabbit anti-human OATP1B3 polyclonal antibody (ab222900, 1:200 dilution; Abcam, UK) at 4 °C overnight. The polyclonal antibody specifically binds to the amino acid sequence (648-695) at the C-terminus of OATP1B3. Subsequently, the slides were incubated with biotin-labeled goat anti-rabbit IgG at room temperature for 15 min. After three washes with PBS, the slides were incubated with horseradish peroxidase-labeled Streptomyces ovalbumin working fluid for 15 min. Finally, the Diaminobenzidine kit (ZLI-9017, Zhongshan Biotechnology, China) was used to visualize the staining reaction, and hematoxylin was used as the nuclear counterstain. Assessment of IHC staining was evaluated independently by two pathologists who did not have prior knowledge about the clinicopathological parameters. Semiquantitative IHC detection was used to determine the OATP1B3 protein levels. The percentage of positive cells was scored as follows: 0 (0%), 1 (1–10%), 2 (11–50%), 3 (51–80%), and 4 (> 80%). The staining intensity was scored as follows: 0 (not stained), 1 (weakly stained), 2 (moderately stained), or 3 (strongly stained). The expression scores were determined by multiplying the percentage of positive cells score with the staining intensity score, and the values ranged from 0 to 12. Finally, the OATP1B3 IHC results were divided into a low expression group (score 0–4) and a high expression group (score ≥ 5) [25].

### RNA extraction and quantitative real-time polymerase chain reaction (qRT-PCR)

TRIzol reagent (TaKaRa, Dalian, China) was used to extract the total RNA from the HCC specimens and adjacent nontumorous tissue samples. The reverse transcription reaction was then carried out on a GeneAmp PCR System 2700 (Applied Biosystems, Singapore) with a PrimeScript RT reagent Kit and a gDNA Eraser (Perfect Real Time) (TaKaRa, Dalian, China). Subsequently, the mRNA expression of OATP1B3 was detected by qPCR on a CFX96 Real-Time System (Thermal Cycler, Bio-Rad, Singapore) using a SYBR Premix Ex Taq II Kit (TaKaRa, Dalian, China). We designed the primers to specifically target the common sequence between lt-OATP1B3 (NM_019844.4) and ct-OATP1B3 (NM_001349920.2). The primer sequences were as follows: OATP1B3 forward 5′-GGAGCAACAGTACGGTCAGT-3′; OATP1B3 reverse 5′-TTCCAGTTGCAACCGTAGGAAT-3′; β-actin forward 5′-CCTGGCACCCAGCACAAT-3′; and β-actin reverse 5′-GGGCCGGACTCGTCATAC-3′. The cycling conditions were as follows: preheating at 96 °C for 5 min; then amplification at 96 °C for 30 s, 57 °C for 30 s, 72 °C for 30 s; cycling 40 times; and last, extension at 72 °C for 10 min. Each experiment was performed in triplicate. β-actin was included as an internal control. The OATP1B3 mRNA expression level was calculated by the 2^−ΔΔCt^ formula.

### Western blotting

Total protein was extracted from tissues by radioimmunoprecipitation assay (RIPA) lysate. The Enhanced BCA Protein Assay Kit (P0010S, Beyotime Biotechnology, China) was used to determine the protein concentration. After quantification, the protein was denatured by boiling. The protein was separated by sodium dodecyl sulfate-polyacrylamide gel electrophoresis (P0012A, Beyotime Biotechnology, China), followed by transfer to polyvinylidene difluoride membranes (Millipore, Burlington, MA, USA). Thereafter, the membranes were blocked at room temperature for 2 h with 5% nonfat dried milk while shaking slowly on a shaker. Membranes were immunoblotted overnight at 4 °C with rabbit anti-human OATP1B3-C-terminal polyclonal antibody (ab139120, 1:250 dilution; Abcam) and β-actin mouse monoclonal antibody (BM0627, 1:1000 dilution; Boster Biological Technology), followed by their respective horseradish peroxidase-conjugated goat anti-rabbit IgG (A0208, 1:1000 dilution; Beyotime Biotechnology, China), and goat anti-mouse IgG (A0216, 1: 1000 dilution; Beyotime Biotechnology, China) antibodies at room temperature for 2 h. The polyclonal antibody specifically binds to the C-terminal peptide sequence of OATP1B3. The membranes were washed three times with Tris-buffered saline with Tween for 10 min each time. The membranes were then detected with enhanced chemiluminescence reagents (Millipore, Burlington, MA, USA). The grayscale values were calculated using the ImageJ software.

### Statistical analysis

All statistical analyses were performed with the SPSS 22.0 software (SPSS, IBM, Chicago, IL, USA). The χ^2^ test or Fisher’s exact test was used to evaluate the correlations between OATP1B3 expression and clinicopathological features. The Kaplan-Meier method and log rank test were used to analyze survival curves. Cox proportional hazard analysis was applied for multivariate analysis. *P* < 0.05 was considered statistically significant.

## Results

### Expression of OATP1B3 in HCC samples

**Fig. 1:**
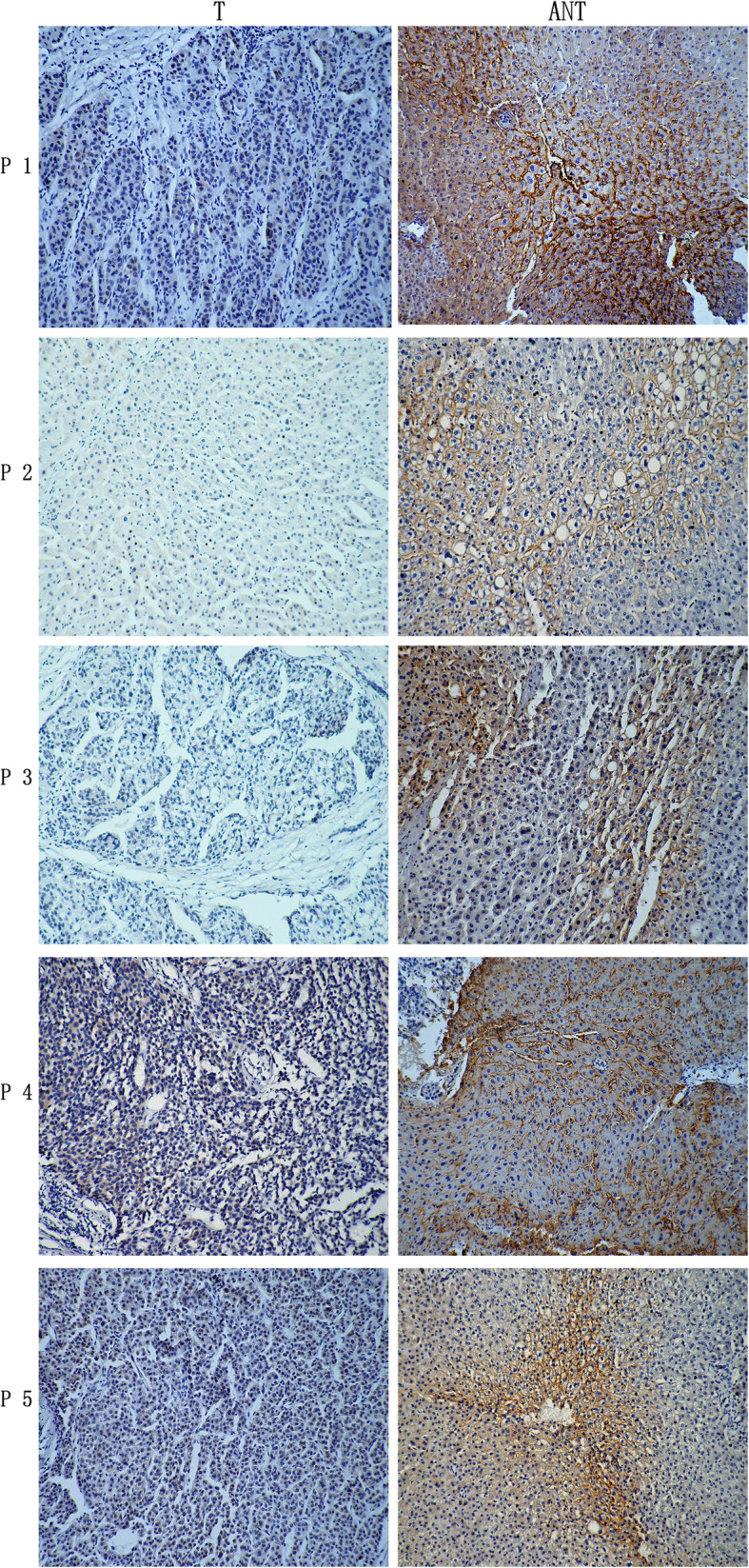
OATP1B3 protein expression in human HCC and matched adjacent nontumorous tissues. Immunohistochemical staining was used to detect the expression of OATP1B3 in 5 pairs of HCC tissues (T) and adjacent nontumorous tissues (ANT) (magnification × 100). *OATP1B3* organic anion transporting polypeptide 1B3, *HCC* hepatocellular carcinoma

**Fig. 2:**
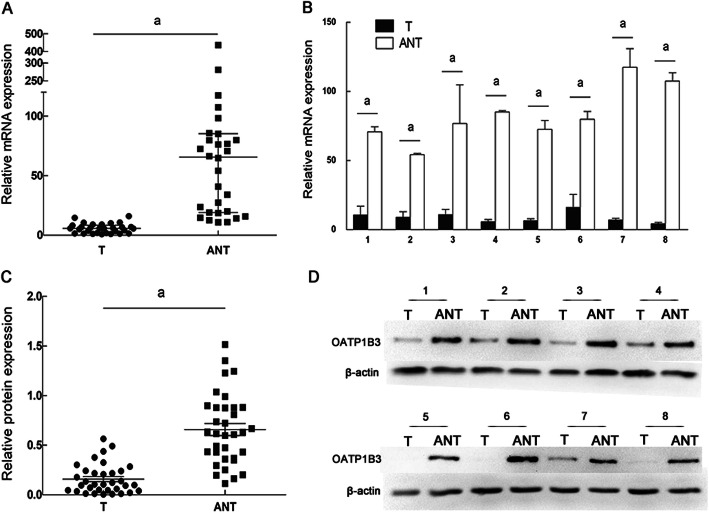
OATP1B3 mRNA and protein expression in HCC tissue and adjacent nontumorous tissues. **a** Relative expression of OATP1B3 was detected by qRT-PCR. OATP1B3 mRNA was higher in matched adjacent nontumorous tissue (ANT) than in HCC tissue (T) (*n* = 30). Relative mRNA expression in the ANT group showed abnormal distribution; thus, the Wilcoxon signed-rank test was applied, and the data are represented as the median ± interquartile range. **b** OATP1B3 mRNA expression levels in 8 pairs of Ts and ANTs were compared. Error bars represent the SD. **c** Relative expression of OATP1B3 protein was detected by Western blotting. OATP1B3 expression in T was significantly lower than that in ANT (*n* = 34). The data are represented as the mean ± SD, and the paired Student’s *t* test was applied. **d** Representative Western blot of OATP1B3 in T and ANT in 8 pairs of samples. Β-Actin was used as an endogenous control. ^*a*^*P* < 0.05

The expression level of OATP1B3 in HCC cancer tissue samples was evaluated according to the aforementioned semiquantitative IHC scoring method. Five representative pairs of tumor and adjacent nontumorous tissue samples with IHC staining are shown in Fig. 1. We found that the OATP1B3 protein was localized to both the plasma membrane and the cytoplasm in tumor tissue samples. Compared with that in HCC specimens (40.5%; 53/131), high expression of OATP1B3 was found in significantly more adjacent nontumorous tissue samples (74.2%; 66/89) (Table 1, *P* < 0.0001). To further verify the reliability of the IHC results, we detected OATP1B3 mRNA expression levels in 30 pairs of randomly selected fresh tumor and adjacent nontumorous tissues using qRT-PCR and protein expression levels in 34 pairs of randomly selected fresh tumor and adjacent nontumorous tissues using Western blotting. The results demonstrated that the expression levels of OATP1B3 mRNA and protein in adjacent nontumorous tissues were significantly higher than those in tumor samples (Fig. 2).

### Correlation between OATP1B3 protein expression and clinicopathological characteristics of HCC

**Fig. 3:**
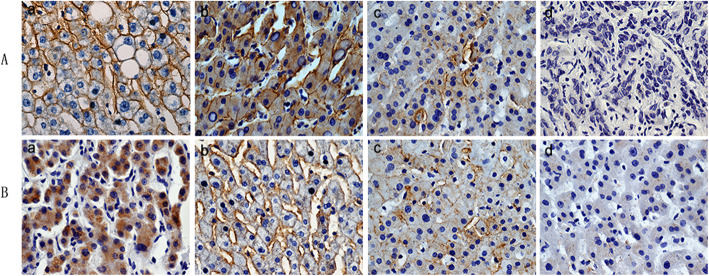
Association between OATP1B3 protein expression and clinicopathological features of HCC. **a** OATP1B3 expression in different degrees of tumor differentiation (a, normal liver tissue; b, well-differentiated; c, moderately differentiated; d, poorly differentiated). **b** OATP1B3 expression in different TNM stages of HCC (a, TNM stage I; b, TNM stage II; c, TNM stage III; d, TNM stage IV)

The χ^2^ test was applied to determine the association between the clinicopathological features of HCC patients and OATP1B3 protein expression. The clinical characteristics of the included patients are shown in Table 1. OATP1B3 protein downregulation was markedly associated with tumor size (*P* = 0.012), TNM stage (*P* < 0.0001), tumor differentiation (*P* < 0.0001) and recurrence (*P* = 0.004). However, there was no significant difference in age, sex, tumor number, HBsAg, cirrhosis, invasion, serum AFP, or tumor capsule status. OATP1B3 expression in normal liver and well-differentiated HCC tissues was significantly higher than that in moderately and poorly differentiated HCC tissues (Fig. 3a). Furthermore, OATP1B3 expression in HCC tissues at TNM III/IV stage was significantly lower than that at TNM I/II stage (Fig. 3b).

### Correlation between OATP1B3 protein expression and clinical outcomes of HCC patients

**Fig. 4:**
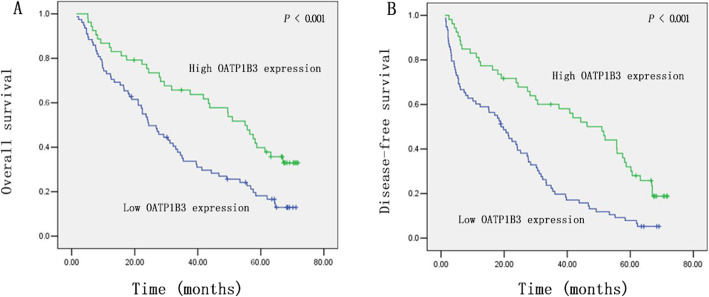
Survival curves for the high OATP1B3 expression group and low OATP1B3 expression group. The log-rank test was applied. **a** Overall survival. **b** Disease-free survival

To determine the prognostic value of OATP1B3 in HCC patients, Kaplan-Meier curves were plotted to compare OS and DFS of HCC patients according to OATP1B3 expression. The log-rank test indicated that patients with low OATP1B3 expression had poorer OS (*P* < 0.001) and DFS (*P* < 0.001) than patients with high OATP1B3 expression (Fig. 4a and b). As shown in Table 2, univariate analysis demonstrated that only OATP1B3 (*P* < 0.001, *P* = 0.001), TNM stage (*P* < 0.001, *P* < 0.001), serum AFP (*P* = 0.045, *P* = 0.037), and invasion (*P* = 0.016; *P* = 0.026) were prognostic indicators of DFS and OS, respectively. Other factors, including sex, age, tumor number, tumor size, tumor differentiation, and tumor capsule status, did not have a significant association with prognosis. We comprehensively analyzed the above four factors affecting prognosis.

**Table 2:** Overall survival and disease-free survival of 131 HCC patients by univariate analysis

Characteristics	*n*	6-year disease-free survival	*P*	6-year overall survival	*P*
OATP1B3			0.000		0.001
Low expression	78	5.3%		12.9%	
High expression	53	18.8%		33.0%	
Age (year)			0.933		0.590
≥ 50	56	9.4%		23.2%	
< 50	75	11.2%		19.8%	
Sex			0.418		0.735
Male	104	10.6%		20.0%	
Female	27	11.1%		25.9%	
Tumor size			0.065		0.205
≥ 5 cm	88	7.8%		17.8%	
< 5 cm	43	15.0%		28.9%	
TNM stage			0.000		0.000
I, II	50	21.3%		32.9%	
III, IV	81	3.5%		13.8%	
Tumor differentiation			0.452		0.076
Well	9	13.0%		50.0%	
Moderate	101	10.5%		15.6%	
Poor	21	10.6%		36.1%	
Serum AFP			0.045		0.037
≥ 20 ng/mL	89	7.3%		18.0%	
< 20 ng/mL	42	16.1%		28.0%	
Invasion			0.016		0.026
Yes	58	6.8%		14.8%	
No	73	13.5%		26.5%	
HBsAg			0.386		0.434
Positive	106	9.2%		20.5%	
Negative	25	17.1%		25.4%	
Tumor number			0.147		0.371
Single	87	12.2%		23.7%	
Multiple	44	7.1%		16.0%	
Liver cirrhosis			0.682		0.972
Yes	91	10.8%		21.7%	
No	40	10.5%		20.8%	
Tumor capsule			0.513		0.785
Yes	45	11.1%		23.6%	
No	86	0.99%		20.2%	

*OATP1B3* organic anion transporting polypeptide 1B3, *TNM stage* tumor node metastasis stage, *AFP* alpha fetoprotein

**Fig. 5:**
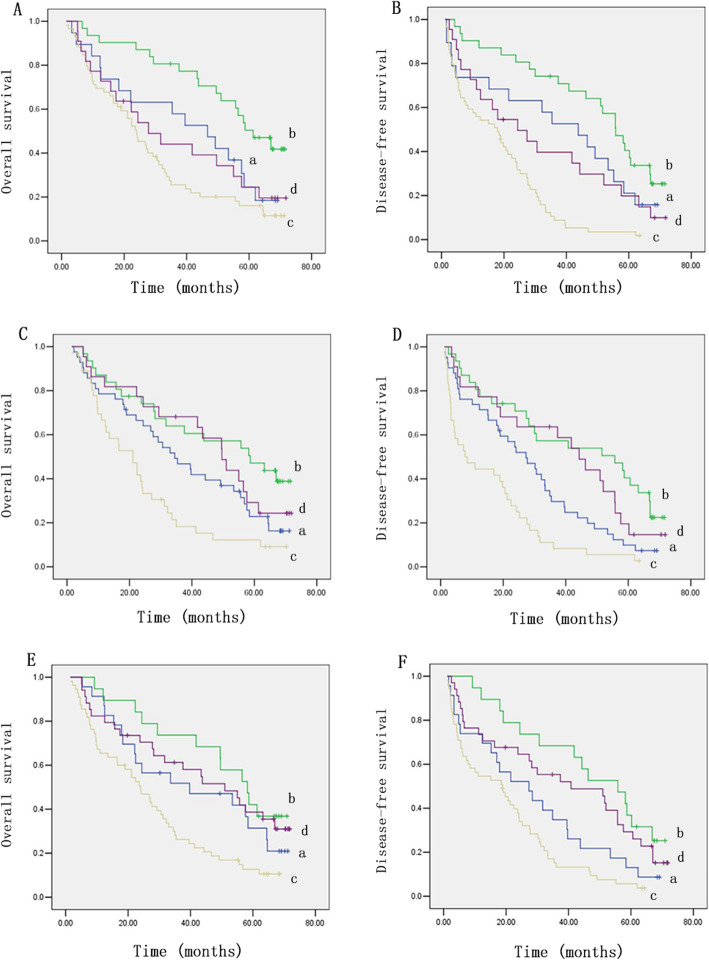
Comprehensive analysis of the survival data. The correlation between combined OATP1B3 expression plus TNM stage and the OS (**a**) and DFS (**b**) of HCC patients. a, low OATP1B3 expression with TNM stages I/II; b, high OATP1B3 expression with TNM stages I/II; c, low OATP1B3 expression with TNM stages III/IV; d, high OATP1B3 expression with TNM stages III/IV. The correlation between combined OATP1B3 expression plus vascular invasion and the OS (**c**) and DFS (**d**) of HCC patients. a, low OATP1B3 expression with no invasion; b, high OATP1B3 expression with no invasion; c, low OATP1B3 expression with invasion; d, high OATP1B3 expression with invasion. The correlation between combined OATP1B3 expression plus serum AFP and the OS (**e**) and DFS (**f**) of HCC patients. a: low OATP1B3 expression with low serum AFP level; b: high OATP1B3 expression with low serum AFP level; c: low OATP1B3 expression with high serum AFP level; d: high OATP1B3 expression with high serum AFP level

The results indicated that the 6-year OS and DFS of 19 (38%) OATP1B3-low HCC patients with TNM stage I/II were significantly lower than those of 31 (62%) OATP1B3-high HCC patients (Fig. 5a and b). Moreover, 59 (72.8%) of the 81 OATP1B3-low HCC patients with TNM stage III/IV had worse outcomes (Fig. 5a and b). Consistent with this, 36 (62.1%) OATP1B3-low patients with vascular invasion had shorter OS and DFS than 22 (37.9%) OATP1B3-high patients (Fig. 5c and d). In contrast, 31 (42.5%) OATP1B3-high HCC patients without vascular invasion had a longer OS and DFS than 42 (57.5%) OATP1B3-low patients (Fig. 5c and d). Similarly, 23 (54.8%) OATP1B3-low patients with low serum AFP had a lower OS and DFS than 19 (45.2%) OATP1B3-high patients (Fig. 5e and f). In addition, 34 (38.2%) OATP1B3-high patients with high serum AFP had better OS and DFS than 55 (61.8%) OATP1B3-low patients with HCC (Fig. 5e and f). These comprehensive analyses revealed that low OATP1B3 expression was related to an increased risk of relapse and death in HCC.

### Cox regression analysis

We incorporated variables (OATP1B3, TNM stage, serum AFP, invasion) with a *P* value < 0.05 from the abovementioned univariate analysis into multivariate Cox proportional hazards analysis. The results indicated that OATP1B3 (*P* = 0.024), invasion (*P* = 0.044), and TNM stage (*P* = 0.021) were significant independent prognostic factors for OS of HCC patients (Table 3). OATP1B3 (*P* = 0.001) and TNM stage (*P* < 0.001) were also significant independent prognostic factors for DFS in HCC patients (Table 3). In summary, OATP1B3 expression may be an independent prognostic factor in HCC patients.

**Table 3:** Overall survival and disease-free survival of HCC patients by multivariate analysis

Variable	Overall survival			Disease-free survival		
	HR	(95% CI)	*P*	HR	(95% CI)	*P*
OATP1B3	0.595	0.379–0.934	0.024	0.508	0.338–0.764	0.001
TNM stage	1.721	1.085–2.728	0.021	2.383	1.569–3.619	0.000
Invasion	1.511	1.010–2.259	0.044	-	-	-

*HR* hazard ratio, *CI* confidence interval, *OATP1B3* organic anion transporting polypeptide 1B3, *TNM stage* tumor node metastasis stage

## Discussion

OATP1B3 is a multispecific transporter that is expressed on the surface of normal liver cells [6, 10]. Because of the low expression of OATP1B3 in HCC [9, 26], it has received little attention from researchers in the past. Many recent studies have demonstrated that OATP1B3 is expressed in various cancer cells and/or tissues [8, 27]. Moreover, OATP1B3 protein expression is highly correlated with the clinicopathological features of cancer patients and has prognostic value [15–17, 28]. Therefore, OATP1B3 may hopefully become a novel biomarker and a target for anticancer therapy in some types of cancers. In this study, we evaluated OATP1B3 expression using IHC staining, qRT-PCR, and Western blotting in HCC tissue and adjacent nontumorous tissue samples at both the transcriptional and translational levels. However, we should emphasize that liver-type (lt)-OATP1B3 or wild-type (wt)-OATP1B3 should be distinguished from cancer-type OATP1B3 (ct-OATP1B3), which is an OATP1B3 mRNA variant and was first reported by Nagai et al. [29] in 2012. In addition, the ct-OATP1B3 protein structure is very similar to that of lt-OATP1B3, which lacks 28 amino acids at the N-terminus [30, 31]. Antibodies used in previous studies bind to the common amino acid region at the C-terminus sequence of OATP1B3, which can recognize both lt-OATP1B3 and ct-OATP1B3 in tissues or cells [14, 16, 22]. In our study, we also used C-terminal antibodies to detect the OATP1B3 protein level. As far as we know, there is no commercially available antibody specific for the N-terminus of ct-OATP1B3. In addition, the primers used to determine the mRNA expression of OATP1B3 were designed against the common sequence between lt-OATP1B3 and ct-OATP1B3. This means that in our study, we detected both lt-OATP1B3 and ct-OATP1B3 at the mRNA and protein levels in HCC specimens and adjacent nontumorous tissues. The results showed that OATP1B3 mRNA and protein were significantly downregulated in HCC tissue samples in comparison with adjacent nontumorous tissue samples, similar to previous reports [9, 26]. It was also found that low OATP1B3 expression was significantly correlated with tumor differentiation, TNM stage, tumor size, and recurrence. This was consistent with previous conclusions that OATP1B3 expression gradually decreases along with tumor dedifferentiation in HCC [18, 32]. This finding may also support Kogita’s and Kim’s findings that Gd-EOB-DTPA-enhanced MRI may predict the histological grade of HCC [33, 34].

Our results presented here show that OATP1B3 was expressed not only on the plasma membrane but also in the cytoplasm in a certain proportion of HCC tissues, which was quite different from the expression of OATP1B3, which was localized predominantly on the plasma membrane in adjacent nontumorous tissues. Thakkar et al. [30] reported that ct-OATP1B3 was localized mainly in the cytoplasm of cancer cells. These results suggested that OATP1B3 expressed in HCC tissues was most likely to be both lt-OATP1B3 and ct-OATP1B3. A previously used OATP1B3 antibody (#651140, Progen Biotechnik, Germany) against the N-terminal sequence that only detects a peptide of lt-OATP1B3 rather than ct-OATP1B3 was applied, and expression was detected in 6 of 22 HCCs (27.3%) [12]. Hence, Imai et al. [35] suggested that lt-OATP1B3 seems to be the predominant OATP1B3 isoform expressed in HCC tissue specimens, which is opposite to the pattern in lung, colon, and pancreatic cancer cells and tissues where ct-OATP1B3 is highly expressed [29]. In human hepatocytes, lt-OATP1B3 mRNA was also the exclusive form, with very low mRNA expression of ct-OATP1B3 [29]. Therefore, we speculated that lt-OATP1B3 may be the primary form expressed, with only a small amount of the ct-OATP1B3 form, in HCC and adjacent nontumorous tissues in our study; this hypothesis warrants further experimental verification.

Furthermore, patients with low OATP1B3 expression had worse OS and DFS than patients with high OATP1B3 expression. These results indicated that OATP1B3 might be involved in suppressing the carcinogenesis and development of HCC. Vasuri et al. [28] reported that OATP1B1/1B3 was inversely associated with the expression of biliary-type keratins K7 and K19 in HCC patients treated with orthotopic liver transplantation (OLT). In addition, patients with OATP1B1/1B3-negative HCCs treated with OLT had significantly lower survival rates after recurrence than patients with OATP1B1/1B3-positive HCCs. Although the antibodies used and patient conditions were different between the two studies, these results still provided some insights into our observations that OATP1B3 may play a pivotal role in the progression of HCC. In our ongoing research, we found that exogenous overexpression of lt-OATP1B3 in HCC cells can promote apoptosis and suppress proliferation (data not shown). Previous reports have shown that OATP1B3 is expressed in various cancer cells, including breast, pancreatic, prostate, colon, lung, and gastric tumors [22]. OATP1B3 and its haplotype-expressing tumors were associated with improved prognosis in breast and prostate cancer [16, 17]. Interestingly, high OATP1B3 expression was also closely correlated with improved 5-year colorectal cancer patient survival within lower grade tumors and earlier stage tumors [15]. By contrast, another study demonstrated that overexpression of OATP1B3 in colorectal cancer cells may provide a survival advantage by interfering with p53 transcriptional activity [14]. Therefore, the effect of OATP1B3 on prognosis in different tumors, even in the same type of tumor, is different or can be opposite. The mechanism underlying these apparently contradictory observations remains unclear. One of the prime reasons is whether the tumors are hormone related, as OATP1B3 can import testosterone, estrogen, and other nutrients into cancer cells. Some experts believe that OATP1B3 may stimulate hormone-dependent cancer cell proliferation and contribute to cancer growth. OATP1B3 can also absorb some anticancer drugs (e.g., sorafenib, methotrexate, docetaxel, paclitaxel) into cancer cells [10]. Thus, some scientists have proposed the activation of anticancer drug delivery pathways and the blockage of hormone delivery paths [36]. In addition, the expression ratio of lt-OATP1B3 to ct-OATP1B3 may have different effects on biological function in different tumors.

A more comprehensive analysis of the survival data suggested that most of the patients with low serum AFP levels or without vascular invasion had OATP1B3-negative tumors and a poor prognosis. Nevertheless, a large number of patients with high serum AFP levels or TNM stage III/IV disease, but with OATP1B3-positive tumors, still had a good prognosis. In multivariate analysis, OATP1B3 (*P* = 0.024), invasion (*P* = 0.044), and TNM stage (*P* = 0.021) were significant independent prognostic factors of OS, while OATP1B3 (*P* = 0.001) and TNM stage (*P* < 0.001) were significant independent prognostic factors of DFS. When evaluating the correlations of patient characteristics with OS and DFS, the *P* values for TNM stage were less than those for OATP1B3 expression, indicating that TNM stage, as an independent prognostic factor has a greater impact on prognosis than OATP1B3. Thus, the survival curve reflected that TNM stage when advanced is totally determining the outcome of the disease. There are many factors considered in TNM staging, especially vascular invasion and lymph node metastasis, distant metastasis, tumor number, and tumor size. It is a comprehensive evaluation and accurately reflects the progression of liver cancer [37]. The 5-year survival rates of patients with TNM stages I, II, and III were 55%, 37%, and 16%, respectively [38]. If cancer cells spread to regional lymph nodes (IVA) or metastasized (IVB), the 5-year survival rates were no more than 12% or 2.5%, respectively [39]. Therefore, with the increase in liver cancer TNM stages, the survival rates of patients decreased rapidly. However, the *P* values of invasion and serum AFP level were greater than those of OATP1B3 in OS and DFS, so the survival curve reflected that high OATP1B3 expression may still indicate a favorable prognosis when combined with the invasion status and serum AFP level. In the present research, serum AFP levels were closely associated with 5-year survival rates in HCC patients, which is consistent with a previous report [40]. Nevertheless, other studies found no correlation between preoperative serum AFP level and postoperative prognosis of HCC patients [41, 42]. Likewise, vascular invasion, which has an adverse effect on prognosis, is representative of the invasiveness of liver cancer cells, but it was not a significant independent prognostic factor of DFS in this study. This is probably because the number of samples was small and the statistical analysis of data may be biased. In addition, the heterogeneity of patients and therapies from different research centers exists among various reports. Overall, these combined analyses revealed that OATP1B3 and other clinicopathological prognostic markers together may constitute an accurate prediction system for evaluating the prognosis of patients with HCC after hepatectomy. Finally, the Cox proportional hazards regression analysis indicated that OATP1B3 may be a promising novel biomarker in HCC patients.

In recent years, research on the expression, regulation, and mechanism of OATP1B3 and its alternative splicing variant in different tumors has emerged. Imai et al. [35] showed that ct-OATP1B3 was strongly expressed in colorectal cancer, cholangiocarcinoma, and pancreatic cancer cells. Due to the remarkable cancer-specific expression profile of ct-OATP1B3 [27, 29], it may represent a promising molecular target for cancer therapy using the herpes simplex virus 1 thymidine kinase-ganciclovir suicide gene system [43]. Nevertheless, ct-OATP1B3 displays defective membrane trafficking and only modest transport activity compared to lt-OATP1B3. Therefore, strategies based on promoting anticancer drug transportation and inhibiting hormone transportation to control cancer growth need to be reassessed in cancers with high ct-OATP1B3 expression [44]. Previous studies reported that DNA methylation of promoter sites and hepatocyte nuclear factor (HNF) 3β affect the expression of OATP1B3, providing a mechanism for downregulated OATP1B3 expression in HCC [26, 35, 45]. In contrast, farnesoid X receptor, HNF1α, HNF1β, and Wnt/β-catenin signaling constitute the transcriptionally activated regulation of OATP1B3 expression [26, 32]. Similarly, an epigenetic-based gene silencing and hypoxia inducible factor-1α (HIF-1α)-dependent mechanism were also found to play important roles in the regulation of ct-OATP1B3 expression in cancer tissues [46]. However, the localization and regulatory mechanisms of lt-OATP1B3 and ct-OATP1B3 in different tumors require further investigation.

This study has some limitations. First, this is a retrospective single-center study with a small sample size, and the results may be biased. A larger multicenter population-based prospective study is necessary to validate the potential prognostic value of OATP1B3 in HCC. Second, we did not detect the expression levels of lt-OATP1B3 and ct-OATP1B3 separately. Further research will be carried out to identify whether lt-OATP1B3 is the predominant OATP1B3 isoform expressed in HCC tissue specimens. Since ct-OATP1B3 research is still at an early stage, the molecular mechanism of ct-OATP1B3 in HCC should also be determined in vivo and in vitro. Third, although tissue biopsy is the most accurate way to obtain molecular information about tumors, it would be more convenient to have information about the circulating levels of molecules, as this information could be more easily used to monitor treated patients. Morio et al. [47] reported that ct-OATP1B3 mRNA was clearly present in extracellular vesicles (EVs) secreted from human colorectal cancer (CRC) cells and could become a promising candidate for a serum-based CRC biomarker. The findings imply the possibility of OATP1B3 detection using serum specimens. A future prospective analysis is needed to validate the expression level of OATP1B3 in HCC patient serum and its prognostic value.

In conclusion, our study demonstrated that low OATP1B3 expression may be a risk factor for relapse and death in patients with HCC after hepatic resection. OATP1B3 may be a promising biomarker for prognosis evaluation and a potential cancer therapy target in HCC patients.

## Data Availability

The data sets during and/or analyzed during the current study are available from the corresponding author on reasonable request.

## Abbreviations

HCC: Hepatocellular carcinoma
OATP1B3: Organic anion transporting polypeptide 1B3
OS: Overall survival
IHC: Immunohistochemistry
DFS: Disease-free survival
Gd-EOB-DTPA: Gadolinium ethoxybenzyl diethylenetriamine pentaacetic acid
MRI: Magnetic resonance imaging
AFP: Alpha fetoprotein
TNM: Tumor node metastasis
PBS: Phosphate-buffered saline
qRT-PCR: Quantitative real-time polymerase chain reaction
RIPA: Radioimmunoprecipitation assay
HR: Hazard ratio
CI: Confidence interval
HNF-3β: Hepatocyte nuclear factor 3β
HIF-1α: Hypoxia inducible factor-1α
OLT: Orthotopic liver transplantation
Wt: Wild-type
Ct: Cancer-type
CRC: Colorectal cancer
EVs: Extracellular vesicles
